# Structural and Functional Insights from the Metagenome of an Acidic Hot Spring Microbial Planktonic Community in the Colombian Andes

**DOI:** 10.1371/journal.pone.0052069

**Published:** 2012-12-14

**Authors:** Diego Javier Jiménez, Fernando Dini Andreote, Diego Chaves, José Salvador Montaña, Cesar Osorio-Forero, Howard Junca, María Mercedes Zambrano, Sandra Baena

**Affiliations:** 1 Colombian Center for Genomic and Bioinformatics from Extreme Environments (GeBiX), Bogotá, Colombia; 2 Departamento de Biología, Unidad de Saneamiento y Biotecnología Ambiental, Pontificia Universidad Javeriana, Bogotá, Colombia; 3 Department of Soil Science, “Luiz de Queiroz” College of Agriculture, University of Sao Paulo, Piracicaba, Brazil; 4 Molecular Genetics and Microbial Ecology Research Groups, Corporación CorpoGen, Bogotá, Colombia; 5 Department of Microbial Ecology, Center for Ecological and Evolutionary Studies (CEES), University of Groningen, Groningen, The Netherlands; J. Craig Venter Institute, United States of America

## Abstract

A taxonomic and annotated functional description of microbial life was deduced from 53 Mb of metagenomic sequence retrieved from a planktonic fraction of the Neotropical high Andean (3,973 meters above sea level) acidic hot spring *El Coquito* (EC). A classification of unassembled metagenomic reads using different databases showed a high proportion of *Gammaproteobacteria* and *Alphaproteobacteria* (in total read affiliation), and through taxonomic affiliation of 16S rRNA gene fragments we observed the presence of *Proteobacteria*, micro-algae chloroplast and *Firmicutes*. Reads mapped against the genomes *Acidiphilium cryptum* JF-5, *Legionella pneumophila* str. Corby and *Acidithiobacillus caldus* revealed the presence of transposase-like sequences, potentially involved in horizontal gene transfer. Functional annotation and hierarchical comparison with different datasets obtained by pyrosequencing in different ecosystems showed that the microbial community also contained extensive DNA repair systems, possibly to cope with ultraviolet radiation at such high altitudes. Analysis of genes involved in the nitrogen cycle indicated the presence of dissimilatory nitrate reduction to N2 *(narGHI, nirS*, *norBCDQ* and *nosZ*), associated with *Proteobacteria*-like sequences. Genes involved in the sulfur cycle (*cysDN*, *cysNC* and *aprA*) indicated adenylsulfate and sulfite production that were affiliated to several bacterial species. In summary, metagenomic sequence data provided insight regarding the structure and possible functions of this hot spring microbial community, describing some groups potentially involved in the nitrogen and sulfur cycling in this environment.

## Introduction

The Colombian Andean region is characterized by high volcanic activity, comprising part of the region called the “Ring of Fire”, and is considered a hotspot for biodiversity [1]. This region has several hot springs that represent unique and extreme ecosystems due to their high elevation and exposure to ultraviolet (UV) light. *El Coquito* (EC) spring is located within the National Natural Park Los Nevados, it has a low pH (2.7) and water temperature of approximately 29°C, which is considerably higher than ambient temperature (∼9°C) and allows us to classify it as a hot spring [2]. A previous analysis of the microbial community at EC hot spring showed that it is dominated by *Bacteria* rather than *Archaea*, with predominance of *Proteobacteria, Firmicutes* and *Planctomycetes*. The planktonic community contained putative chemotrophic bacteria potentially involved in cycling of ferrousiron and sulfur-containing minerals and phototrophic organisms (mostly eukaryotic micro-algae) [3].

Microbial diversity in hot springs is dictated by environmental physicochemical characteristics (pH, redox potential, temperature and concentration of trace elements) [4]–[6]. In acidic hot springs the most representative genera described are *Acidithiobacillus*, *Acidimicrobium*, *Sulfobacillus, Thiomonas*, *Leptospirillum* and *Hydrogenobaculum* [7], [8]. These chemolithotrophic acidophiles are often the predominant primary producers and may also contribute to iron and sulfur cycling via oxidization of reduced inorganic sulfur and ferrous iron compounds [9]. Other acidic hot springs with mesophilic temperature (30–35°C) are dominated by *Acidiphilium* mesophilic heterotrophs and *Acidithiobacillus* autotrophic thermotolerant sulfur oxidizers [10]. In extremely acidic and UV light-irradiated environments, primary production may also be mediated by mesophilic phototrophic acidophiles (mainly eukaryotic micro-algae) [11]. Many of these studies have assessed microbial diversity by 16S rRNA gene analysis [12]–[14], which is useful but does not provide information on ecologically relevant genes involved in various biogeochemical cycles.

Metagenomic analyses using high throughput sequencing or library construction have been extremely valuable for describing microbial structure and functionality in extreme ecosystems [15]–[17] and for identifying novel genes [18]–[20]. Comparative metagenomic studies have also characterized microbial communities and shown differences in functionality in several ecosystems [21], [22]. The current and most frequently used tools for taxonomic and functional classification of metagenomic reads are based on local alignments (BLAST) against different databases and associating best hits to taxa, specific genes, functional identifiers or metabolic pathways. However, a more comprehensive picture of the genes and functions in a metagenomic dataset can be obtained using different algorithms, parameters and databases for read assignment [23].

In this work we analyzed the sequences obtained from a metagenome of the EC high mountain (Paramo ecosystem) acidic spring to obtain a deeper view of the genes present and the functional-based structure of the microbial community in the planktonic fraction. The taxonomic and functional profile obtained from metagenomic unassembled reads differed depending on the database used. The microbial community was dominated by *Proteobacteria* (*Gammaproteobacteria* and *Alphaproteobacteria*), with some sequences mapping within the genomes of the acidophiles *Acidiphilium cryptum* and *Acidithiobacillus caldus*, a finding that broadens the repertoire of natural environments where these organism are found. A more in depth analysis of the nitrogen and sulfur cycles using KEGG pathways to associate best hits to taxa and specific genes showed some of the processes involved in denitrification, nitrogen fixation, and sulfide oxidation, the latter likely linked to the acidity of the environment.

## Materials and Methods

### Ethics Statement

The studied locations are in a state owned National Park. The study did not involve endangered or protected species. All necessary permits were obtained for the described field studies from the corresponding national authorities, MAVDT (contract number 15, 2008) for access to genetic resources and UAESPNN (research permit code DTNO-N-20/2007).

### Sample collection and processing

**Figure 1:**
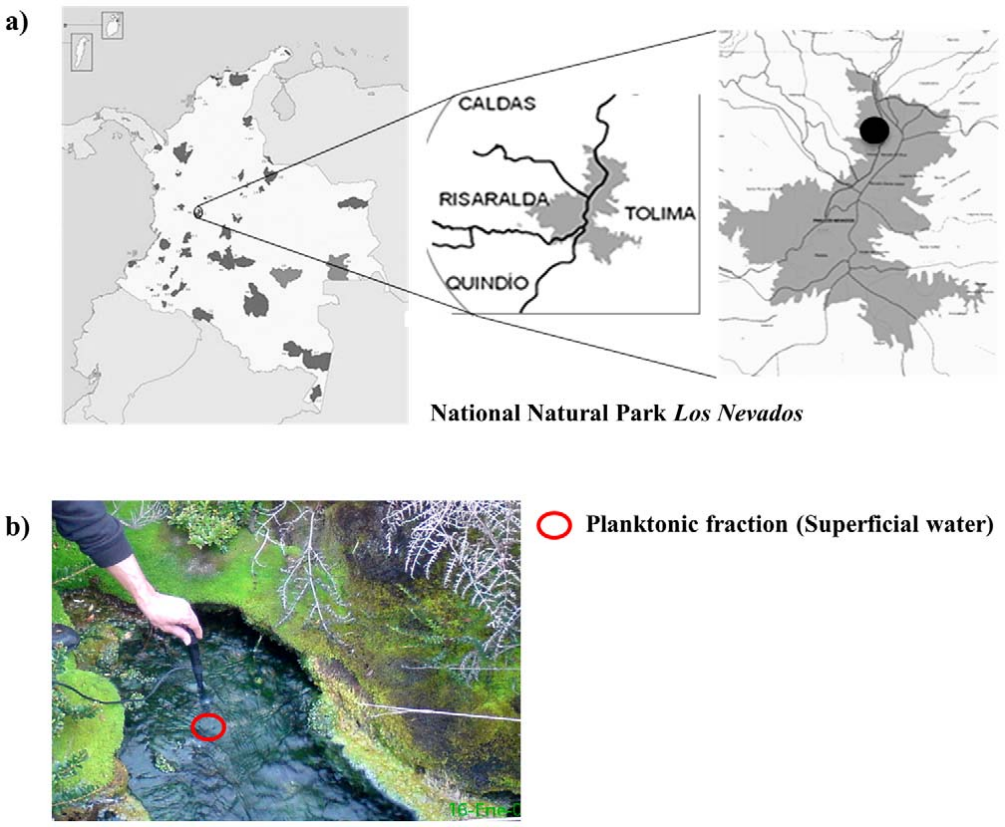
Location of EC hot spring in the National Natural Park Los Nevados. a) Geographical location. b) Photographs of the acidic hot spring *El Coquito* (EC), circle indicates the planktonic fraction.

Surface water (15 L) was collected in separate sterile plastic containers for biological and physicochemical analyses in April 2008 (rainy season) at EC hot spring, located at 3,973 meters above sea level (masl) (04°52′27′′ N; 75°15′51.4′′ W) in the National Natural Park Los Nevados (Figure 1a). Due to the difficulty in accessing this location, the samples were kept and transported at 4°C to the laboratory and processed within 18 h for further physicochemical analysis (SO_4_^2−^, Ca^2+^, Mg^2+^, Na^+^, K^+^, Fe^2+^, Fe^3+^ CaCO^3+^, NO^3+^, Chloride, PO^4+^ and total dissolved solids) and DNA isolation [3]. Temperature and pH were recorded *in situ* using a Hach pH-meter equipped with a pH and temperature probe.

### DNA extraction and pyrosequencing

Water (10 L) was filtered through 5.0 µm cellulose filters (Fisherbrand Q5), to remove particles and large cells, and then through 0.22 µm polycarbonate filters (GTTP, Millipore). Planktonic cells on filters were processed to obtain the DNA, as previously described [3]. A total of 20 ng of metagenomic DNA was amplified with pHi29 polymerase using the isothermal multiple displacement amplification (MDA) system (REPLI-g, Qiagen) by incubating at 30°C for 1.5 hrs. This step was monitored for contamination using a negative control tube without DNA. The reaction was stopped by heating at 65°C for 3 min, and the final product was purified using UltraClean GelSpin DNA Extraction Kit (MoBio Laboratories Inc., Carlsbad, CA, USA), resulting in 46.2 µg of DNA (∼1,850 ng/µl). A total of 12.2 µg of metagenomic DNA was used for library preparation using emulsion PCR and pyrosequencing using 454 GS FLX Titanium technology on ½ plate (Engencore, University of South Carolina, Columbia, SC, USA). The total reads obtained (292,559) from a SSF (standard flowgram file) were filtered and trimmed based on length and quality using an in-house python script (www.corpogen.org/tools/clean454.zip). Sequences with a minimum length of 30 bp were evaluated using a sliding window of 20 bp and only those sequences with a recommended quality score of ≥20, were retained [24], [25]. All sequences are available in the metagenomic RAST (MG-RAST) server under project ID 4449206.3.

### Taxonomic assignment of metagenomic sequences

The taxonomic assignment of the unassembled metagenomic dataset was performed using BLASTX [26] on MG-RAST v3.0 [27] against the GenBank (NCBI-nr), RefSeq and SEED databases using a cut-off E-value of 1e-10 and minimum alignment of 50 bp. WebCARMA v1.0 online system [28] was also used with previously proposed parameters [23], [24]. SSU rRNA (16S rRNA) reads were extracted from the dataset using a HMMER search against a hidden Markov model built based on multiple sequence alignments [29], aligned using the NAST align tool (batch size for NAST: 5; minimum percentage identity: 75) (http://greengenes.lbl.gov/cgi-bin/nph-NAST_align.cgi), and taxonomically classified using the classification tool for aligned SSU rRNA sequences (http://greengenes.lbl.gov/cgi-bin/nph-classify.cgi), which has been shown to have the highest accuracy for assigning taxonomy to short pyrosequencing reads when compared with other methods [30].

### Recruitment plots to draft genomes from acidophilic bacteria

A fragment recruitment plot of the EC hot spring metagenome was performed against the draft microbial genomes of *A. cryptum* JF-5 (349163.4), *Legionella pneumophila* str. Corby (400673.6) and *A. caldus* (33059.1), using BLASTX in the MG-RAST v2.0 platform. The criteria for counting a hit were: cut-off E-value of 1e-5, minimum alignment length of 50 bp and minimum identity of 40%.

### Functional analysis using COG and KEGG identifiers

A functional-based classification of EC metagenome was conducted using BLASTX and RPS-BLAST (cut-off E-value of 1e-10) against the COG database [31] downloaded from the NCBI ftp site and the NCBI (nr/nt) local database. Annotation results for BLASTX against the NCBI-nr database were loaded into the MEtaGenome ANalyzer software (MEGAN v4.0) and classified using KEGG identifiers [32], according to suggested parameters for the Lowest Common Ancestor algorithm (LCA) (maximum number of match per read: 5; min support: 5; min score: 35; and top percent: 10) [33].

### Metabolic mapping of energy metabolism

The allocation of reads into metabolic maps was obtained using BLASTX (cut-off E-value of 1e-10) against the NCBI-nr database and analyzed with the MEGAN v4.0 software. The metabolic pathways involved in nitrogen and sulfur transformations were recognized using KEGG identifiers. The number of reads in each pathway (oxidative phosphorylation, methane metabolism, nitrogen metabolism, carbon fixation pathways in prokaryotes, and carbon fixation in photosynthetic organism, sulfur metabolism and photosynthesis) were recorded. Extracted sequences were given taxonomic assignments by performing BLASTX against the NCBI-nr and RefSeq databases, and analyzed with the MEGAN v4.0 software and the MG-RAST v3.0 server.

### Comparison with other metagenomes

We performed a taxonomic and functional comparison of the EC hot spring metagenome using BLASTX (cut-off E-value of 1e-10) against the RefSeq, COG, KEGG and SEED databases. Comparisons were carried out with different datasets obtained by pyrosequencing in different ecosystems: acid and non-acid mines, coral reefs, marine waters, tropical forest soil, Andean forest acid soil and pristine mangrove sediment (Table 1). A double hierarchical dendrogram was created using the Bray-Curtis distance metric and normalized values in the MG-RAST v3.0 server.

**Table 1:** Characteristics of datasets used for comparative metagenomic analysis.

	RSM (4440281.3)	BSM (4440282.3)	POCR (4440039.3)	SW (4443702.3)	PO (4443713.3)	TFS (4446153.3)	HAFS (4445417.3)	PMS (4451036.3)	AHEC (4449206.3)
**Number of reads**	334,386	388,627	351,205	209,073	221,744	782,404	619,288	217,605	280,753
**Average size read (bp)**	105±17	99±16	105±17	226±60	239±55	411±103	310±118	222±107	190±95
**Total Mbp**	35.5	38.5	37.0	47.2	53.0	322.2	192.3	48.5	53.5
**Mean GC content (%)**	49±12	44±10	46±10	40±9	39±9	59±6	62±7	54±10	52±10
**Taxonomy classified reads (%) ^a^**	0.0068	0.038	0.021	47.5	55.3	59.4	58.7	33.5	8,7
**% KEGG matches ^b^**	0.0095	0.055	0.037	19.8	23.1	16.9	19.5	12,0	1,8
**% SEED matches ^b^**	0.0185	0.108	0.100	47.6	55.7	37.6	43.7	29.0	4.2
**Reference**	[39]	[39]	[80]	[81]	Unpublished	[82]	Unpublished	[24]	This study

RSM: Red Soudan Mine (acidic); BSM: Black Soudan Mine; POCR: Pacific Ocean (coral reefs); SW: Sea Water; PO: Pacific Ocean; TFS: Tropical Forest Soil; HAFS: High Andean Forest Soil; PMS: Pristine Mangrove Sediments; AHEC: Acidic Hot Spring EC. ^a^ Using RefSeq database (Bacteria, Archaea, Eukarya and Virus) (cut-off E-value 1e-10); ^b^ cut-off E-value 1 e-10.

## Results and Discussion

In this study, we carried out a metagenomic analysis (taxonomic and functional-basis assignment of metagenomic unassembled reads) of the planktonic microbial community present in EC hot spring located in the Colombian Andes (Figure 1). The target hot spring is surrounded by endemic vegetation and characterized by an acidic pH (2.7), high solar radiation (approximately 9–11 mW/cm^2^ nm UV-B) [34], high mineral content (1,003 mg SO_4_^2−^ L^−1^, 320 mg Ca^2^ L^−1^, 56.6 mg Cl^−^ L^−1^, 8.27 mg of total iron L^−1^ and 45.2 mg Na^+^ L^−1^) and high total dissolved solids (2,280 mg L^−1^). As a complement to a previous 16S rRNA gene analysis [3], and in order to assess the microbial community structure using direct sequencing, the metabolic pathways were evaluated and the major players involved in nitrogen and sulfur transformations were identified. Total metagenomic DNA eluted from the filter was equivalent to 116 ng of bacterial DNA per liter, which could be indicative of a low cell density [35], and is consistent with the low planktonic biomass detected previously [3]. Due to the low amount of recovered metagenomic DNA (580 ng), amplification was performed using phi29 polymerase prior to 454 pyrosequencing, using the appropriate controls (negative) recommended by the manufacturers to diminish the risk of contamination from the starting material [36]. So far this is the most advanced and reliable method available to achieve these descriptions from very limited initial metagenomic DNA [37]. From a total of 292,559 sequences obtained, 280,753 metagenomic reads with an average size of 190±95 bp (equivalent to 53 Mb of DNA) were retained after quality assessment and trimming using an in-house python script. The amount of data falls within what has been reported for some other high throughput sequencing datasets: hot spring (12 Mb) [38], acid mine (35 Mb) [39], canine fecal sample (53 Mb) [21], mangrove sediments (215 Mb) [24] and Antarctic permafrost (388 Mb) [40]. In our dataset most sequences (>90%) had a GC content between 45–60% (mean of 52±10%). An additional 85,409 sequences failed to pass the Quality Control (QC) in MG-RAST pipeline. Of the sequences that passed QC, 28,304 sequences (10.1%) contained predicted proteins with known functions and 131,697 sequences (46.9%) contained predicted proteins of unknown function.

### Taxonomic assignment of metagenomic sequences

**Figure 2:**
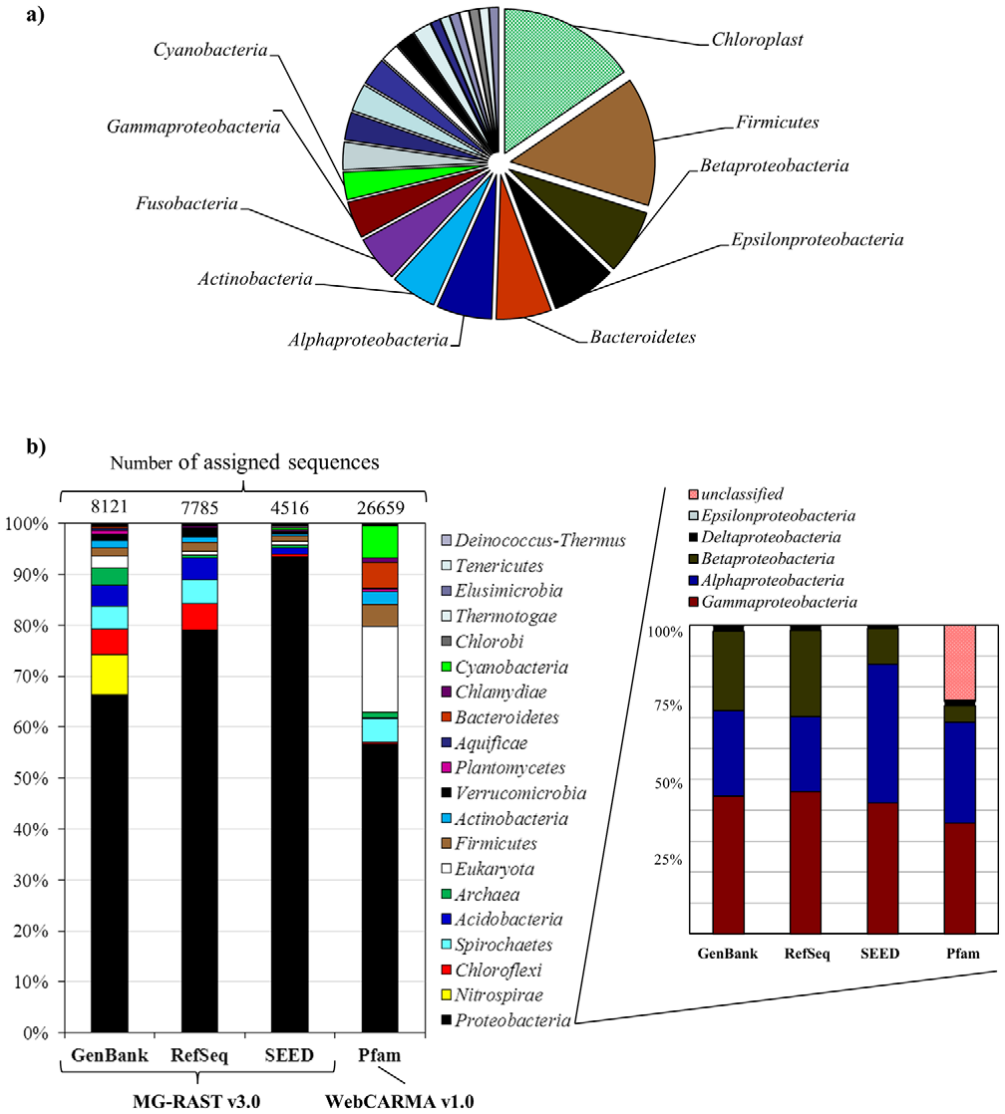
Classification of metagenomic reads from EC hot spring. a) Taxonomic affiliation based on 97 SSU rRNA extracted sequences using the Greengenes database; b) Comparison of the taxonomic assignment of metagenomic sequences, based on predicted proteins using the MG-RAST v3.0 server and WebCARMA v1.

To provide a framework for the metagenomic analyses, we first compared the taxonomic assignments obtained by retrieving SSU rRNA reads and by using BLASTX to classify total reads. Using the hidden Markov model approach, 97 SSU rRNA sequences were identified within the metagenomic dataset. These SSU rRNA sequences, which are free from PCR and cloning biases, showed a prevalence of *Proteobacteria* (*Gammaproteobacteria* > *Alphaproteobacteria* > *Betaproteobacteria*) (∼25%), followed by micro-algae chloroplast ribosomal DNA (∼15%), *Firmicutes* (∼14%) and *Bacteroidetes* (∼6%) (Figure 2a; Supplementary Table S1). These results are consistent with the previous characterization of this site based on clone library and pyrotag sequence data [3]. The low number of SSU sequences recovered (0.03% in our metagenomic dataset) is consistent with other reports [22], [24]. This could be due to drawbacks with sequence assignment using short sequence reads (<100 bp) [41], to secondary structure conformations [42], problems due to low coverage using the 454 platform, absence of assembly or possible MDA biases [43]. These results also highlight the need to develop more refined bioinformatics tools to precisely and efficiently extract sequence reads belonging to the gene family of interest [44], [45].

In addition to SSU rRNA gene analysis, a taxonomic affiliation was also carried out in the MG-RAST server using all the metagenomic reads to obtain a broad idea of the groups present in this ecosystem. This analysis showed differences in the number of sequences classified depending on the database used: 2.9% (8,121 reads) for the GenBank database, followed by RefSeq with 2.7% (7,785 reads) and SEED with 1.6% (4,516 reads). With WebCARMA (Pfam database) we assigned 26,659 reads (9.4%) (Figure 2b). These low values contrast with the Amazon River metagenome reads classified using BLASTX against NCBI-nr in MG-RAST, 49% of 1.1 million pyrosequencing reads [46]. These low values indicate that the EC hot spring could contain a great amount of newly described sequences, as suggested by the high number of unclassified 16S rRNA pyrotags reported previously [3], or could be explained by a large amount of noncoding DNA present in the genomes (especially in micro-*Eukaryotes*). A major bias using this approach is that the current databases contain mostly sequences from cultivable microorganisms. In addition, the accuracy also depends on the representation of the different taxonomic groups in a database [15]. Thus, up to 90% of the sequences in a metagenomic dataset may remain unidentified due to the lack of reference sequences [47]. Hopefully, sequence analysis and taxonomic affiliation will improve as more genome sequencing projects become available and bioinformatics software tools are developed [48]–[50].

The predominant phylum in all databases, detected by total read assignment using BLASTX, was *Proteobacteria* (∼76%). This was followed by *Nitrospirae* (10%), which was assigned only in the GenBank database, *Chloroflexi*, *Spirochaetes* and *Acidobacteria* (Figure 2b). The highest percentage of sequences assigned to *Eukarya* (13%) was obtained using WebCARMA, which also assigned 7% of the sequences to the phylum *Cyanobacteria* (Figure 2b). These latter sequences most probably correspond to micro-algae, as it was observed previously for this ecosystem [3], or could be indicating that this group of oxygenic phototrophs could be present both in planktonic and in mat communities [51]. The fact that few phototrophic bacteria (*Chlorobi*, *Cyanobacteria*, and *Chloroflexi*) were detected in EC hot spring, both in this and the previous study based on 16S rRNA analysis [3], is consistent with the notion that *Cyanobacteria* are sensitive to metals and solutes found in acidic waters [52]. The eukaryotic micro-algae detected might play important roles in primary production by using solar energy at the surface, similar to what occurs in surface acid streamers and other acidic extreme environments [53]. A Pearson analysis showed limited correlation (0.73) between taxonomic affiliation based on SSU rRNA (Greengenes database) and total metagenomic read assignment (RefSeq database), which could be partially explained by the fact that most of phylotypes identified by SSU rRNA sequences belonged to linages for which genome sequences are not yet available [44], [54], or by variation in SSU rRNA copies and/or genome size [55].

**Figure 3:**
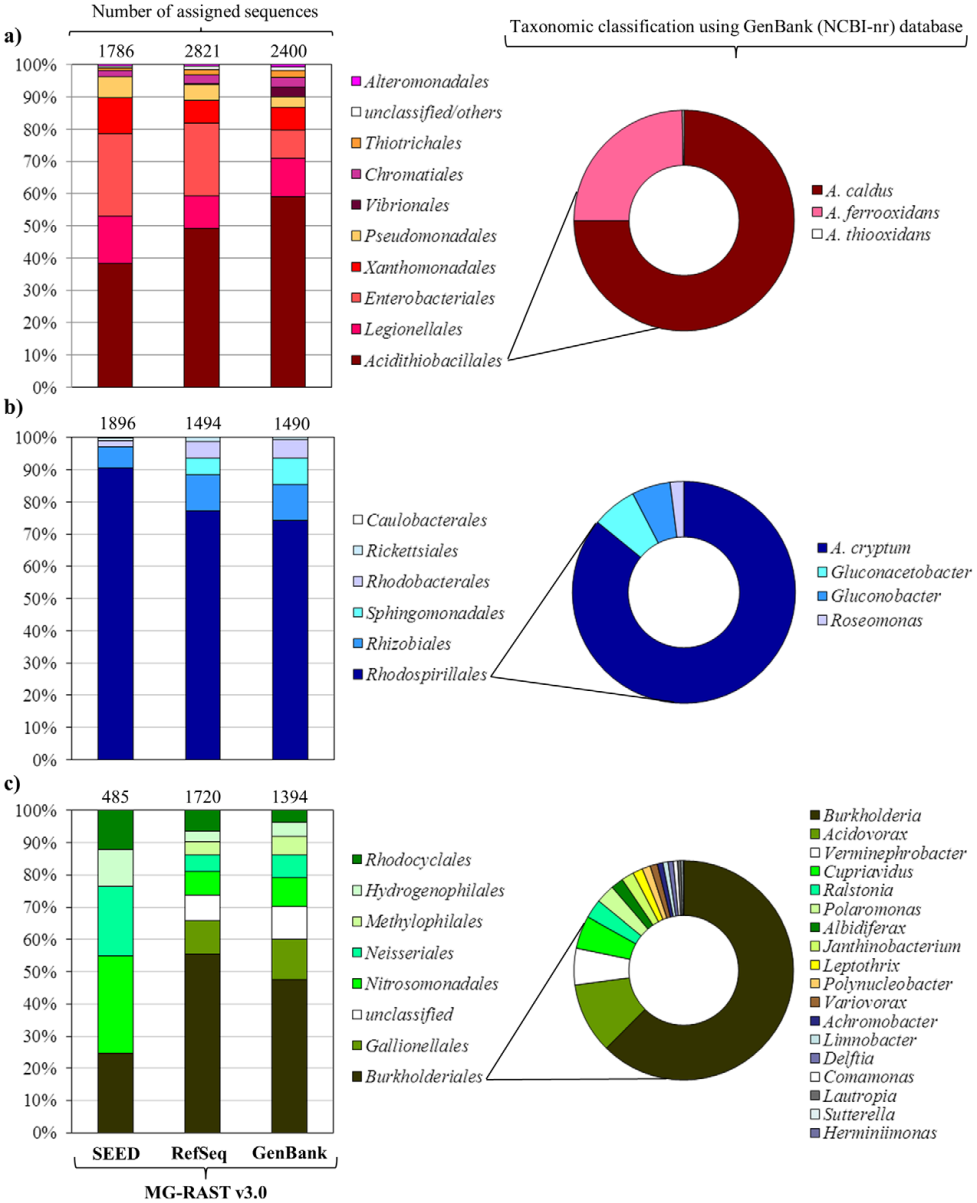
Taxonomic comparison within *Proteobacteria* of total metagenomic sequences based on predicted proteins using BLASTX against different databases in MG-RAST v3.0 server. a) *Gammaproteobacteria* b) *Alphaproteobacteria* c) *Betaproteobacteria*.

*Gammaproteobacteria* was the dominant annotated class (∼40%) in all databases (based on predicted proteins). Using the RefSeq and GenBank databases we observed a high number of sequences related to *Acidithiobacillales* (∼54%) (represented by sequences related to *Acidithiobacillus caldus*, *Acidithiobacillus ferroxidans* and *Acidithiobacillus thiooxidans*), followed by *Legionellales* (Figure 3a). *Acidithiobacillus*, which have been found in extremely acidophilic sulfur-oxidizing biofilms “snottites”, can grow on reduced inorganic sulfur and iron compounds as the energy sources [56] and oxidize sulfur via sulfide–quinone reductase and the sox pathway [17]. In other environments, these microorganisms play an important role in sulfur mobilization by catalyzing metal sulfide oxidation at low pH and by releasing sulfate into solution [57]. Several species of infectious and non-infectious Legionella have been found using molecular techniques in hot springs, acid mines and rivers [58]–[61]. *Alphaproteobacteria* was the second most dominant class (∼30%), with sequences related to the order *Rhodospirillales* (∼80%) (Figure 3b) that included *A. cryptum* (1,681 and 1,017 assigned reads using SEED and GenBank, respectively). *Acidiphilium*, one of the most abundant and versatile genera found in acid mine drainage [60], represents autotrophic and heterotrophic, sulfur oxidizing mesophilic bacteria able to grow at pH between 2.5–6.0 [62]. *Acidiphilium* species can also carry out photosynthesis using Zn-BChl (photopigments) [63], which may be advantageous for growth and survival in oligotrophic environments. Sequences assigned to *Betaproteobacteria* (∼20%) included the orders *Burkholderiales* (between 25–55%), reported in warm and hot acid-sulfate springs [64], [65], followed by *Gallionellales*, *Nitrosomonadales*, *Rhodocyclales* and *Hydrogenophilales* (Figure 3c). Despite the short length of reads and the possibility of compositional bias associated with sample handling, these results provide a general view of the taxa involved and, interestingly, are consistent with the previous study of the diversity in this ecosystem based on SSU rRNA analysis, which showed the predominance of the orders *Burkholderiales*, *Rhodocyclales*, *Legionellales*, *Rhodospirillales*, *Clostridiales*, *Plantomycetales* and *Nitrospirales* [3].

### Comparison with draft genomes from acidophilic bacteria

The reads for predicted proteins in our dataset were next compared to genomes belonging to taxa that contained a large number of affiliated reads, such as *A. cryptum, A. caldus* and *Legionella* sp. (Figure 3). Of the total metagenomic reads compared to the *A. cryptum* JF-5 genome, 1,063 reads were mapped to 312 of 3,612 features (putative genes) (Supplementary Table S2). Some of the genes that mapped in highest proportion and with lowest E-value (less than 1e-50) encoded enzymes involved in transposition and integration of mobile genetic elements (transposases). Genes that encoded phage portal proteins were identified, indicating the presence of *A. cryptum* phages or prophages, as has been found in the Amazon River metagenome [46] and in acidophilic microbial communities [66]. The presence of genes potentially involved in lateral gene transfer might indicate that horizontal exchange of genetic material could be a common occurrence in EC hot spring, as has been suggested for hydrothermal chimneys [16], [67]. Other sequences that mapped to *A. cryptum* genes were involved in energy production, amino sugar metabolism, fatty acid biosynthesis, RNA synthesis, as well as ATP carrier proteins, ABC transporters, hydrolytic enzymes and hypothetical proteins. The metagenomic reads were also compared with the *L. pneumophila* and the *A. caldus* genomes. Of the total metagenomic reads, 624 mapped to 447 out of 3,423 features from the *L. pneumophila* str. Corby genome, and 365 reads mapped into the *A. caldus* genome (Supplementary Table S2). In the *L. pneumophila* genome sequences mapped to genes associated with various processes such as posttranslational modification and RNA synthesis, among others, and to a sequence that encodes a helicase responsible for regulating RNA synthesis at low temperatures [68]. Finally, in the *A. caldus* genome, we observed sequences associated with transposases (as occurred for *A. cryptum*) and histone-like mobilization proteins.

### Functional analysis using COG, KEGG and SEED identifiers

**Figure 4:**
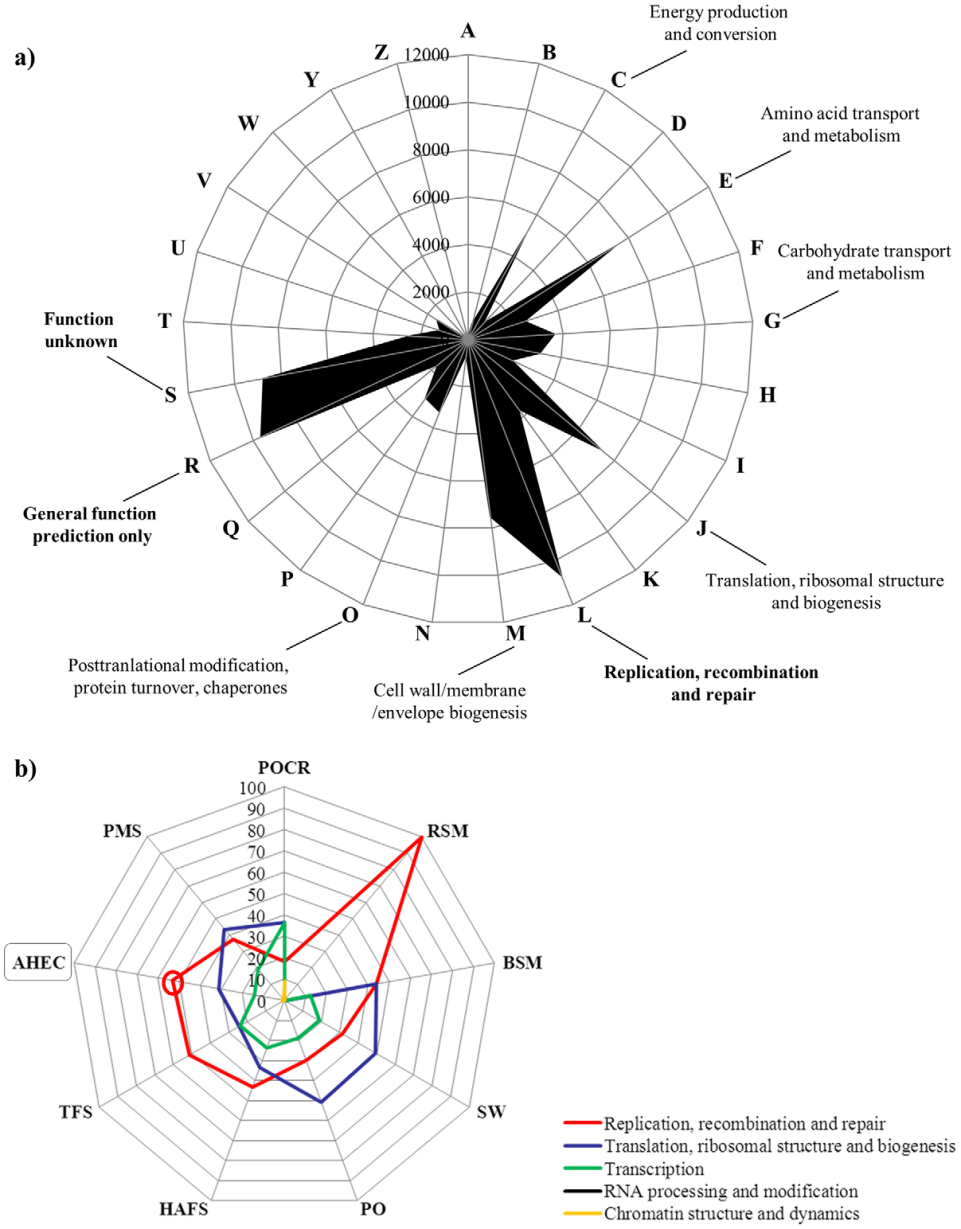
Functional assignment using BLASTX against the COG database. a) Analysis of metagenomic sequences obtained from EC hot spring, the numbers indicate the amount of sequences affiliated to the predominant COG identifiers. b) Comparison of various metagenomes (RSM: Red Soudan Mine; BSM: Black Soudan Mine; POCR: Pacific Ocean (coral reefs); SW: Sea Water; PO: Pacific Ocean; TFS: Tropical Forest Soil; HAFS: High Andean Forest Soil; PMS: Pristine Mangrove Sediments; AHEC: Acidic Hot Spring EC) using the percentage of annotated reads in the specified COG categories.

The proportion of matches to the COG database exceeded the KEGG and SEED matches, similar to previous reports [22], [24]. A total of 87,023 reads (30.9%) were assigned to 25 COG categories and most of the sequences were related to replication, recombination and repair (L) (10,712 reads, ∼12%) (Figure 4a). Some of the other COG categories identified included general function prediction only (R) (9,637 reads), function unknown (S) (8,777 reads) and energy production and conversion (C) (5,001 reads) (Figure 4a). Using BLASTX against the NCBI-nr database and the MEGAN software, 19,876 sequences (∼7.0%) were associated with KEGG pathways, specifically to metabolism of carbohydrates (2,623), amino acids (2,584), energy (1,920) and nucleotides (1,431) (Supplementary Figure S1). Some functions were found using both COG and KEGG identifiers, such as DNA repair, translation, transcription, replication, homologous recombination and carbohydrate and energy metabolism while others, such as photosynthesis, regulation and cell signaling, metabolism (protein, sulfur, RNA), carbohydrates and respiration were identified using the SEED database (Supplementary Figure S2b). Even though there might be biases associated with the sampling strategy and with MDA, suggesting that these functions may be important in this ecosystem. The presence of recombination, replication and repair systems, that were evident in the COG analysis and by comparison with other metagenomes (Figure 4b), could also be important in this ecosystem where high UV radiation (at ∼4000 masl), acidic pH and high water temperature may cause significant damage to DNA. Reports of enrichment for genes involved in mismatch DNA repair and homologous recombination in deep sea hydrothermal vent chimneys and hot springs suggest that the microbial communities have evolved extensive DNA repair systems to cope with extreme conditions that have potential deleterious effects on their genomes [51], [16]. Finally, in this study we also identified sequences associated with *quorum sensing* and cellular communication in biofilms, which could form on the surface of the EC hot spring (Figure 1b).

### Energy metabolism and mapping of nitrogen and sulfur transformations

**Figure 5:**
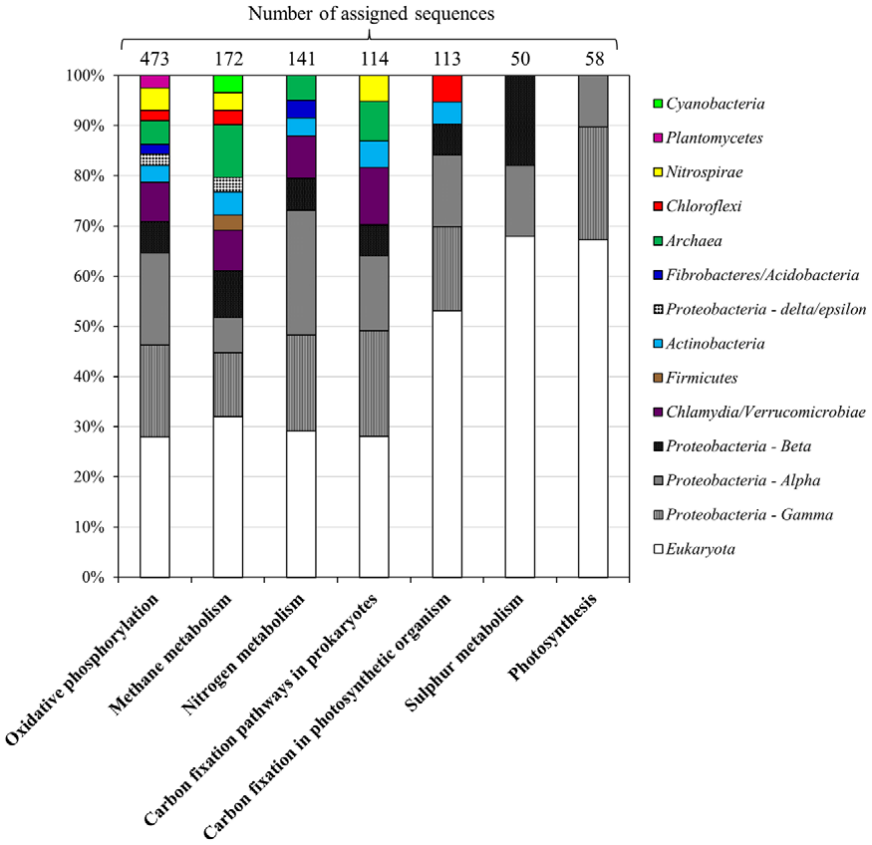
Taxonomic assignment of metagenomic reads obtained from EC hot spring related to energy metabolism (KEGG identifiers). The taxonomic affiliation was performed by BLASTX against NCBI-nr and analyzed using the MEGAN v 4.0 (LCA algorithm), which provides phylogenetic classification at different levels, depending on the sequence read. Classification is shown at the kingdom level for *Archaea* and *Eukaryota*; the phyla shown are from *Bacteria* and only for *Proteobacteria* were reads classified to the class level: *Alpha* (*Alphaproteobacteri*a), *Beta* (*Betaproteobacteria*), *Gamma* (*Gammaproteobacteria*), *delta/epsilon* (*Deltaproteobacteria*/*Epsilonproteobacteria*).

To gain more insight regarding possible functions in this community, we looked at reads associated with different metabolic pathways. A total of 1,920 reads were mapped to energy metabolism using BLASTX against the NCBI-nr database, and corresponded to both *Eukarya* and *Bacteria*. Most of the bacterial reads were assigned to *Alphaproteobacteria*, *Gammaproteobacteria* and *Betaproteobacteria*. Sequences involved in various pathways, such as oxidative phosphorylation, methane, nitrogen, and carbon fixation, were associated with *Chlamydia/Verrucomicrobia*, *Actinobacteria*, *Nitrospirae* and *Archaea*. Also, reads associated with *Planctomycetes*, *Cyanobacteria* and *Chloroflexi* were observed in oxidative phosphorylation, methane metabolism and carbon fixation in photosynthetic organisms, respectively (Figure 5).

We also looked more closely at pathways involved in nitrogen and sulfur metabolism, since these may be important in habitats where terminal electron acceptors other than O_2_ may be important, such as nitrate, ferric iron, arsenate, thiosulfate, elemental S, sulfate or CO_2_. A total of 268 and 127 sequences were associated with nitrogen and sulfur metabolism, respectively. Approximately 80% of the genes associated with the nitrogen cycle were related to *Alphaproteobacteria*, *Gammaproteobacteria*, *Betaproteobacteria* and *Eukarya*. A detailed analysis using the MEGAN software showed the presence of *narGHI* genes (14 reads) (dissimilatory reduction of nitrate) (Table 2; Supplementary Figure S3a). Based on current models of dissimilatory nitrate reduction in bacteria, a nitrite reductase (*nirK* or *nirS*) would be required to produce NO, which serves as a substrate for nitric oxide reductase (*norB*) to produce N_2_O [38]. Several sequences from EC spring were associated with *nirS* and *norBDQ*, with *nosZ* (associated with magnetotactic bacteria), which is important for the conversion of N_2_O to N_2_, and 13 sequences were associated with ferredoxin-nitrite reductase (*nirA*). The balance among these pathways is influenced greatly by environmental conditions, such as temperature, pH, oxygen, nitrate availability, and organic matter content. Finally, we also identified *nifK* (associated with sulfate reducing *Thermodesulfovibrio* and sulfur reducing bacteria *Desulfitobacterium*), a gene involved in the synthesis of molybdenum dependent (Mo-dependent) nitrogenase, suggesting that in addition to denitrification, nitrogen fixation could also be taking place in EC hot spring [69], [70] (Table 2; Supplementary Figure S3a). Enzymes such as ammonium monooxygenase subunit A and glutamine synthetase were not detected in our dataset, due either to the low depth of sequencing achieved or to the fact that they may not be present in EC hot spring, similar to what has been reported previously in Yellowstone hot springs [38]. Based on the taxonomic affiliation of the identified reads, we propose that dissimilatory nitrate reduction is most likely carried out by *Proteobacteria*-like organisms and assimilatory reduction of nitrate is carried out mostly by acidophilic micro-algae, *Acidobacteria*, *Spartobacteria* and *Alphaproteobacteria* (Table 2; Supplementary Figure S3a).

**Table 2:** Sequences associated with specific functions and taxa within the nitrogen cycle using KEGG pathways.

Number of reads assigned	E.C Number	Orthology KEGG	Enzyme name	Gene	Organism	Class	E-value ^c^
14	1.7.99.4	K00370	nitrate reductase 1, alpha subunit	*nar*G	*Pseudomonas stutzeri*	*Gammaproteobacteria*	1 e–25
					*Thiomonas itermedia*	*Betaproteobacteria*	4 e–41
					*Desulfococcus oleovorans*	*Deltaproteobacteria*	8 e–27
		K00371	nitrate reductase 1, beta subunit	*nar*H	*Thiomonas* sp.	*Betaproteobacteria*	6 e–35
					*Oligotropha* *carboxidovorans*	*Alphaproteobacteria*	1 e–68
							1 e–26
		K00374	nitrate reductase 1, gamma subunit	*nar*I	*Pseudomonas fluorescens*	*Gammaproteobacteria*	1 e–12
4	1.3.12.16	K00459	nitronate monooxygenase		Candidatus *Koribacter versatilis*	*Acidobacteria*	7 e–16
					*Legionella pneumophila*	*Gammaproteobacteria*	6 e–18
10	1.7.99.7	K04561	nitric-oxide reductase, cytochrome b-containing subunit I	*nor*B	*Legionella longbeachae*	*Gammaproteobacteria*	1 e–16
					*Legionella pneumophila*		2 e–20
							2 e–14
		K02164	nitric-oxide reductase NorE protein	*nor*E	*Agrobacterium tumefaciens*	*Alphaproteobacteria*	8 e–46
		K02305	nitric-oxide reductase, cytochrome c-containing subunit II	*nor*C	*Bradyrhizobium* sp.	*Alphaproteobacteria*	1 e–14
		K04748	nitric-oxide reductase NorQ protein	*nor*Q	*Thiobacillus denitrificans*	*Betaproteobacteria*	1 e–54
2	1.7.99.6	K00376	nitrous-oxide reductase	*nos*Z	*Magnetospirillum magneticum*	*Alphaproteobacteria*	6 e–28
					*Cardiobacterium hominis*	*Gammaproteobacteria*	3 e–15
7	1.18.6.1	K02586	nitrogenase molybdenum- iron protein alpha chain	*nif*D	*Bradyrhizobium* sp.	*Alphaproteobacteria*	7 e–24
		K02591	nitrogenase molybdenum- iron protein beta chain	*nif*K	*Desulfitobacterium* *hafniense*	*Firmicutes*	8 e–31
					*Thermodesulfovibrio yellowstonii*	*Nitrospira*	3 e–18
4	1.7.1.4	K00362	nitrite reductase (NAD(P)H) large subunit	*nir*B *	*Sphigomonas* sp.	*Alphaproteobacteria*	1 e–18
7	1.7.7.1	K00366	ferredoxin-nitrite reductase	*nir*A *	Candidatus *Koribacter versatilis*	*Acidobacteria*	1 e–18
					*Acidobacterium* *capsulatum*	*Acidobacteria*	7 e–26
					*Pseudochlorella* sp. ^a^	*Trebouxiophyceae*	3 e–15
					*Thalassiosira pseudonana* ^b^	*Coscinodiscophyceae*	3 e–15
					*Chthoniobacter flavus*	*Spartobacteria*	2 e–18
2	1.7.2.1	K00368	nitrite reductase	*nir*S	*Nitrosococcus halophilus*	*Gammaproteobacteria*	5 e–36

* assimilatory nitrate reduction; ^a^ micro-algae; ^b^ diatom: ^c^ (cut-off E-value 1e -10).

Most of the genes involved in the sulfur cycle were related to the conversion of sulfate into adenylylsulfate and to the further generation of sulfite and H_2_S (Supplementary Figure S3b), although, we also observed sequences related to serine O-acetyltransferase production. Genes were identified for cysteine synthase AB (*cysKM*) and for formation of adenylysulfate (Table 3; Supplementary Figure S3b). We also detected a bi-functional enzyme (sulfate adenylytransferase and adenylysulfate kinase) (*cysNC*). In dissimilatory sulfate reduction and sulfur oxidation, adenosine-5′-phosphosulfate (APS) reductase (Apr) is considered a key enzyme. In the sulfate-reducing pathway, sulfate has to be activated to APS by ATP-sulfurylase at the expense of ATP, Apr converts the APS to sulfite, and then, sulfite is reduced to sulfide by dissimilatory sulfite reductase (Dsr) [71]. The alpha subunits of Apr enzymes are found in all known sulfate reducing and most of sulfur oxidizing prokaryotes. We detected genes involved in conversion of adenylylsulfate to sulfite (*aprAB*; *cysH*), in sulfite reduction and H_2_S formation (*cysI*), and in the oxidation of sulfite to sulfate (sulfite oxidase enzyme) (Table 3; Supplementary Figure S3b).

**Table 3:** Sequences associated with specific functions and taxa within the sulfur cycle using KEGG pathways.

Number of reads assigned	E.C Number	Orthology KEGG	Enzyme name	Gene	Organism	Class	E-value ^e^
8	2.3.1.30	K00640	serine O-acetyltransferase	*cys*E	*Gemmata obscuriglobus*	*Planctomycetacia*	6 e–36
					*Bryantella formatexigens*	*Clostridia*	1 e–32
37	2.7.7.4	K00956	sulfate adenylyltransferase subunit 1	*cys*N	*Laribacter hongkongensis*	*Betaproteobacteria*	2 e–15
					*Stigmatella aurantiaca*	*Deltaproteobacteria*	6 e–11
					*Bacterium Ellin514*	*Verrucomicrobiae*	3 e–11
		K00957	sulfate adenylyltransferase subunit 2	*cys*D	*Clostridium cellulovorans*	*Clostridia*	2 e–24
					Uncultured Archeon	*Archaea*	2 e–18
					*Rhodomicrobium vannielii*	*Alphaproteobacteria*	4 e–10
					*Opitutaceae bacterium*	*Opitutae*	6 e–20
					*Marinobacter aquaeolei*	*Gammaproteobacteria*	2 e–19
					*Clostridium cellulovorans*	*Clostridia*	7 e–16
		K00958	sulfate adenylyltransferase	cysDN	*Leptospirillum ferrodiazotrophum*	*Nitrosospira*	1 e–75
					*Micromonas pusilla* ^a^	*Prasinophyceae*	2 e–21
					*Planctomyces maris*	*Planctomycetacia*	4 e–29
					*Thialkalivibrio* sp.	*Gammaproteobacteria*	5 e–26
					*Rhodothermus marinus*	*Sphingobacteria*	5 e–23
					*Silicibacter lacuscaerulensis*	*Alphaproteobacteria*	2 e–17
					*Syntrophobacter fumaroxidans*	*Deltaproteobacteria*	2 e–18
					*Thiobacilllus denitrificans*	*Betaproteobacteria*	6 e–15
		K00955	bifunctional enzyme CysN/CysC	*cys*NC	*Acidiphilium cryptum*	*Alphaproteobacteria*	7 e–72
					*Xenorhabdus nematophila*	*Gammaproteobacteria*	6 e–41
8	1.8.3.1	K00387	sulfite oxidase *	X	*Phaeodactylum tricornutum* ^b^	*Bacillariophyceae*	1 e–14
					*Nematostella vectensis* ^c^	*Anthozoa*	8 e–15
9	1.8.99.2	K00395	adenylylsulfate reductase, subunit B	*apr*B	*Thiobacillus aquaesulis*	*Betaproteobacteria*	3 e–32
					*Thermodesulfovibrio yellowstonii*	*Nitrosospira*	5 e–45
		K00394	adenylylsulfate reductase, subunit A	*apr*A	*Thiobacilllus denitrificans*	*Betaproteobacteria*	9 e–24
					*Desulfirivibrio alkaliphilus*	*Deltaproteobacteria*	3 e–14
					*Thiobacilllus denitrificans*	*Betaproteobacteria*	2 e–22
13	2.7.1.25	K00955	bifunctional enzyme CysN/CysC	*cys*NC	*Acidiphilium cryptum*	*Alphaproteobacteria*	7 e–72
					*Xenorhabdus nematophila*	*Gammaproteobacteria*	6 e–41
		K00860	adenylylsulfate kinase	*cys*C	*Geobacter sp.*	*Deltaproteobacteria*	7 e–15
					*Burkholderia sp.*	*Betaproteobacteria*	3 e–15
					*Chloroflexus sp.*	*Chloroflexi*	8 e–22
					*Chlamydomonas reinhardtii* ^a^	*Chlorophyceae*	1 e–24
							4 e–33
							1 e–32
23	2.5.1.47	K12339	cysteine synthase B	*cys*M	*Thioalkalivibrio sp.*	*Gammaproteobacteria*	5 e–16
					*Streptomyces clavuligerus*	*Actinobacteria*	4 e–24
		K01738	cysteine synthase A	*cys*K	*Desulfirivibrio alkaliphilus*	*Deltaproteobacteria*	3 e–12
					*Chlamydomonas reinhardtii* ^a^	*Chlorophyceae*	2 e–13
					*Thalassiosira pseudonana* ^b^	*Coscinodiscophyceae*	5 e–16
					*Gemmata obscuriglobus*	*Planctomycetacia*	3 e–12
					*Chlamydomonas reinhardtii* ^a^	*Cholorophyceae*	5 e–22
					*Polytomella parva* ^a^	*Cholorophyceae*	1 e–15
					*Symbiobacterium thermophilum*	*Clostridia*	2 e–15
							2 e–15
4	1.8.4.8	K00390	phosphoadenosine phosphosulfate reductase	*cys*H	*Acidobacterium capsulatum*	*Acidobacteria*	4 e–22
10	1.8.1.2	K00381	sulfite reductase (NADPH) hemoprotein beta-component	*cys*I	*Haliangium achraceum*	*Deltaproteobacteria*	2 e–24
					*Azoarcus sp.*	*Betaproteobacteria*	5 e–30
					*Citomicrobium bathyomarinum*	*Alphaproteobacteria*	1 e–32
		K00380	sulfite reductase (NADPH) flavoprotein alpha-component	*cys*J	*Chlamydomonas reinhardtii* ^a^	*Chlorophyceae*	1 e–17
3	1.8.7.1	K00392	sulfite reductase (ferredoxin)	*sir*	*Physcomitrella patens* ^d^	*Embryophyta*	1 e–13
					*Micromonas pusilla* ^a^	*Prasinophyceae*	2 e–12

* sulfur oxidation; ^a^ micro-algae; ^b^ diatom; ^c^ sea anemone; ^d^ moss; ^e^ (cut-off E-value 1e -10).

### Comparison with other metagenomes

**Figure 6:**
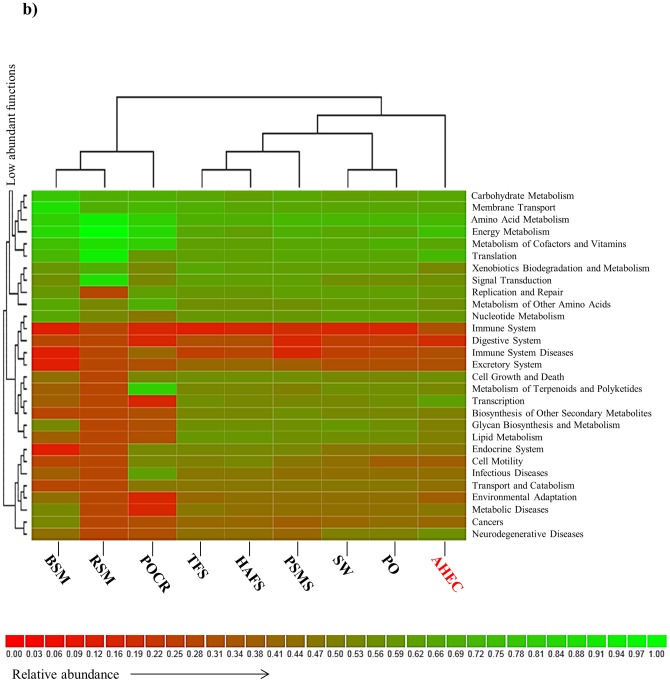
Functional clustering based on total reads of various metagenomes (RSM: Red Soudan Mine; BSM: Black Soudan Mine; POCR: Pacific Ocean (coral reefs); SW: Sea Water; PO: Pacific Ocean; TFS: Tropical Forest Soil; HAFS: High Andean Forest Soil; PMS: Pristine Mangrove Sediments; AHEC: Acidic Hot Spring EC). The data was compared to KEGG databases. Dendrogram linkages are based on relative abundance of the metabolic identifiers (KEGG database) within the samples.

In order to uncover unique features and highlight relevant taxa or possible microbial functions of the EC metagenome, we compared the dataset from EC hot spring against a collection of selected metagenomes obtained by pyrosequencing (to minimize potential biases because of differences in sequence length) (Table 1). The taxonomic composition of the nine metagenomes done using BLASTX against the RefSeq database, showed differences at the phylum level and a closer association of EC hot spring with ocean metagenomes (except coral reefs) (Supplementary Figure S2a). This result was consistent with a hierarchical functional comparison based on KEGG and SEED identifiers (Figure 6; Supplementary Figure S2b). In soil samples and in mangrove sediments, *Acidobacteria*, *Verrucomicrobia* and *Planctomycetes* were found in a high relative proportion. Interestingly, EC hot spring had a higher proportion of sequences related with *Streptophyta*, *Bacillariophyta*, unclassified sequences derived from *Eukarya*, *Cyanobacteria*, *Chloroflexi*, *Spirochaetes*, *Acidobacteria*, *Euryarchaeota* and *Nitrospirae*, when compared with other metagenomes (Figure 6a). In particular, the metagenome from EC differed in processes related to energy metabolism, translation, photosynthesis, cell signaling and replication-repair (Figure 6b; Figure 4b).

### Concluding remarks and future perspectives

In this metagenomic study, the taxonomic and functional features (metabolic pathways) of the microbial community present in a Colombian acidic hot spring EC were analyzed. Unexplored and extreme ecosystems such as this have been previously shown to be of interest as a source of new biotechnological products [72], [73], species and ecological features (as homologous recombination in speciation processes) [74]–[76]. Only a small proportion of the sequences in EC hot spring had matches against the available databases, suggesting that there is a high proportion of novel DNA sequences, consistent with analysis of extreme environments using pyrosequencing data and possibly due to a high proportion of novel taxa, as suggested by previous analysis based on 16S rRNA pyrotags [3], or to the limited length of reads and sequence depth achieved. The taxa identified both by classification based on SSU read affiliation and total read assignment (annotated portion of the microbial community), such as *Acidiphilium*, *Burkholderia*, *Acidovorax*, *Acidithiobacillus*, *Nitrosospira*, *Legionella*, *Thiobacillus, Desulfitobacterium* and acidophilic micro-algae were comparable to those identified based on PCR amplification of 16S rRNA genes [3]. The annotated portion of the microbial community indicated the presence of DNA repair systems that may be involved in homologous recombination and adaptation processes to extreme environments. The identification of genes coding for nitrogen and sulfur cycling indicate a population involved in dissimilatory-assimilatory reduction of nitrate, and conversion of sulfate into adenylylsulfate and sulfite. Future studies will target the comparison between metatrascriptomic and metagenomic analysis [77]–[79], structure dynamics of microbial communities (especially micro-eukaryotes), and analysis of other pathways or ecological processes, like the carbon cycle, photosynthesis and the oxidation or reduction of iron, which can lead to further understanding of these communities. Overall, this sequence-based exploration of the metagenomic content in an Andean hot spring goes beyond the identification of taxa using 16S rRNA gene analysis and provides insight into both taxonomical composition and metabolic potential. However a greater depth of sequencing will be required to more fully assess the functional diversity present in this ecosystem, the association of metabolic routes with particular taxa and their relevance to community dynamics.

## Supporting Information

Figure S1 **Functional assignment of metagenomic sequences using BLASTX against NCBI-nr. The data was analyzed using MEGAN v4.0 software and KEGG identifiers.**
(PPT)

Figure S2 **Taxonomic and functional clustering based on total reads of various metagenomes (RSM: Red Soudan Mine; BSM: Black Soudan Mine; POCR: Pacific Ocean (coral reefs); SW: Sea Water; PO: Pacific Ocean; TFS: Tropical Forest Soil; HAFS: High Andean Forest Soil; PMS: Pristine Mangrove Sediments; AHEC: Acidic Hot Spring EC).** The data were compared to a) RefSeq and b) SEED databases. Dendrogram linkages are based on relative abundance of the phylum level (RefSeq) and relative abundance of the metabolic identifiers (Subsystems, SEED database) within the samples.
(PPT)

Figure S3 **Partial a) nitrogen and b) sulfur pathways identified by KEGG affiliation of the sequences from EC hot spring.** Boxes indicate the KEGG characteristic identified and numbers in gray circles indicate the amount of sequence reads affiliated to the KEGG function.
(PPT)

Table S1 **Phylogenetic affiliation of SSU rRNA sequences.** The aligned reads were classified taxonomically using “classify a batch of sequences against multiple taxonomies tool” from Greengenes.
(XLS)

Table S2 **Metagenomic reads obtained from EC hot spring that mapped to the** ***A. cryptum*** **JF-5,** ***L. pneumophila*** **str. Corby and** ***A. caldus*** **draft genomes.**
(XLS)
