# Supplementary material for: Structural and Functional Insights from the Metagenome of an Acidic Hot Spring Microbial Planktonic Community in the Colombian Andes
Source: PLoS One. 2012 Dec 14;7(12):e52069. doi: 10.1371/journal.pone.0052069 (PMC3522619; doi:10.1371/journal.pone.0052069)
Supplement: Figure S1 — Functional assignment of metagenomic sequences using BLASTX against NCBI-nr. The data was analyzed using MEGAN v4.0 software and KEGG identifiers. (PPT) [file pone.0052069.s001.ppt]

## Slide 1
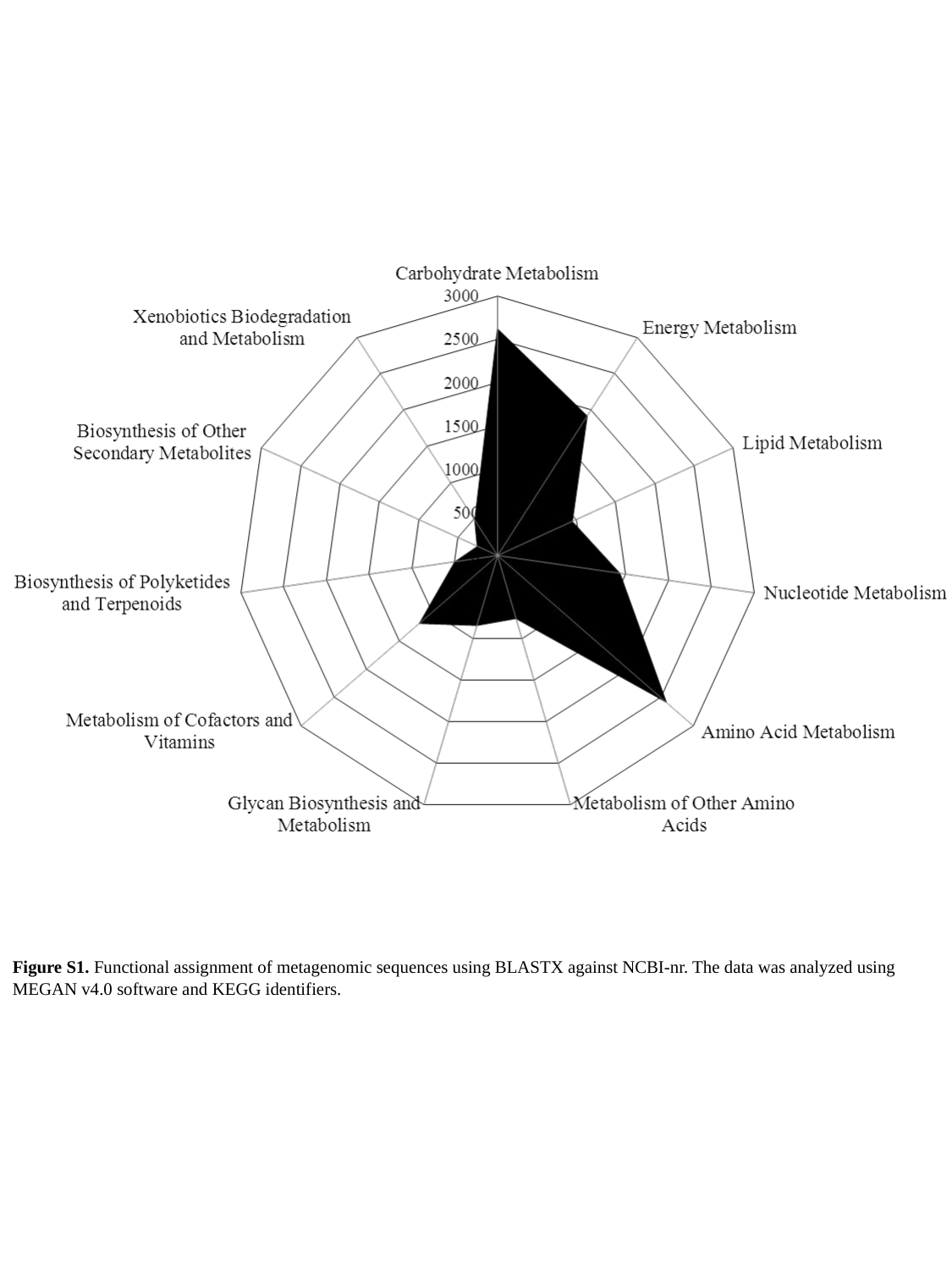

Figure S1. Functional assignment of metagenomic sequences using BLASTX against NCBI-nr. The data was analyzed using MEGAN v4.0 software and KEGG identifiers.
