# Supplementary material for: Structural and Functional Insights from the Metagenome of an Acidic Hot Spring Microbial Planktonic Community in the Colombian Andes
Source: PLoS One. 2012 Dec 14;7(12):e52069. doi: 10.1371/journal.pone.0052069 (PMC3522619; doi:10.1371/journal.pone.0052069)
Supplement: Figure S2 — Taxonomic and functional clustering based on total reads of various metagenomes (RSM: Red Soudan Mine; BSM: Black Soudan Mine; POCR: Pacific Ocean (coral reefs); SW: Sea Water; PO: Pacific Ocean; TFS: Tropical Forest Soil; HAFS: High Andean Forest Soil; PMS: Pristine Mangrove Sediments; AHEC: Acidic Hot Spring EC). The data were compared to a) RefSeq and b) SEED databases. Dendrogram linkages are based on relative abundance of the phylum level (RefSeq) and relative abundance of the metabolic identifiers (Subsystems, SEED database) within the samples. (PPT) [file pone.0052069.s002.ppt]

## Slide 1
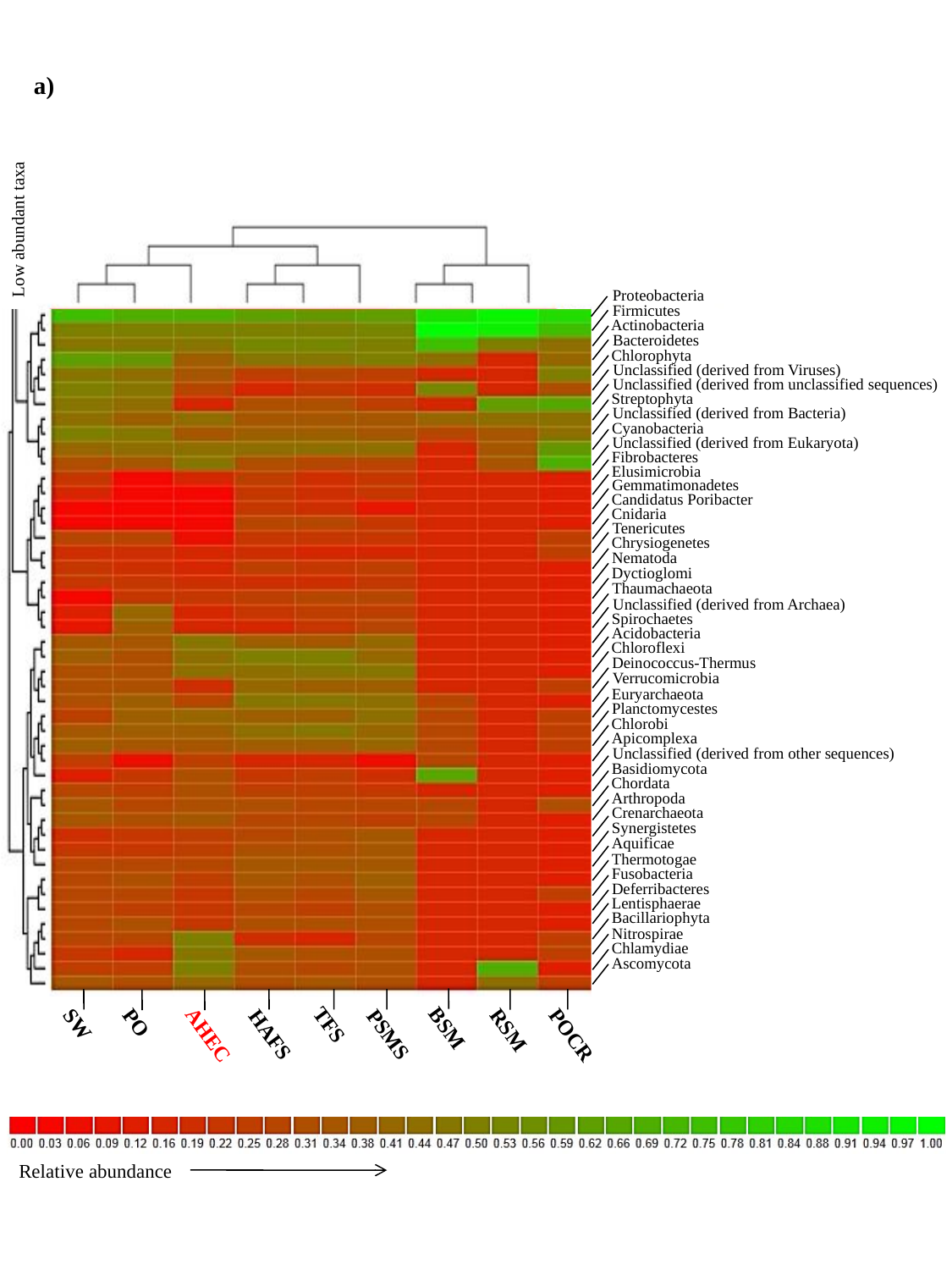

a)
Low abundant taxa
Proteobacteria
Firmicutes
Actinobacteria
Bacteroidetes
Chlorophyta
Unclassified (derived from Viruses)
Unclassified (derived from unclassified sequences)
Streptophyta
Unclassified (derived from Bacteria)
Cyanobacteria
Unclassified (derived from Eukaryota)
Fibrobacteres
Elusimicrobia
Gemmatimonadetes
Candidatus Poribacter
Cnidaria
Tenericutes
Chrysiogenetes
Nematoda
Dyctioglomi
Thaumachaeota
Unclassified (derived from Archaea)
Spirochaetes
Acidobacteria
Chloroflexi
Deinococcus-Thermus
Verrucomicrobia
Euryarchaeota
Planctomycestes
Chlorobi
Apicomplexa
Unclassified (derived from other sequences)
Basidiomycota
Chordata
Arthropoda
Crenarchaeota
Synergistetes
Aquificae
Thermotogae
Fusobacteria
Deferribacteres
Lentisphaerae
Bacillariophyta
Nitrospirae
Chlamydiae
Ascomycota
PO
SW
TFS
BSM
RSM
HAFS
PSMS
POCR
AHEC
Relative abundance

## Slide 2
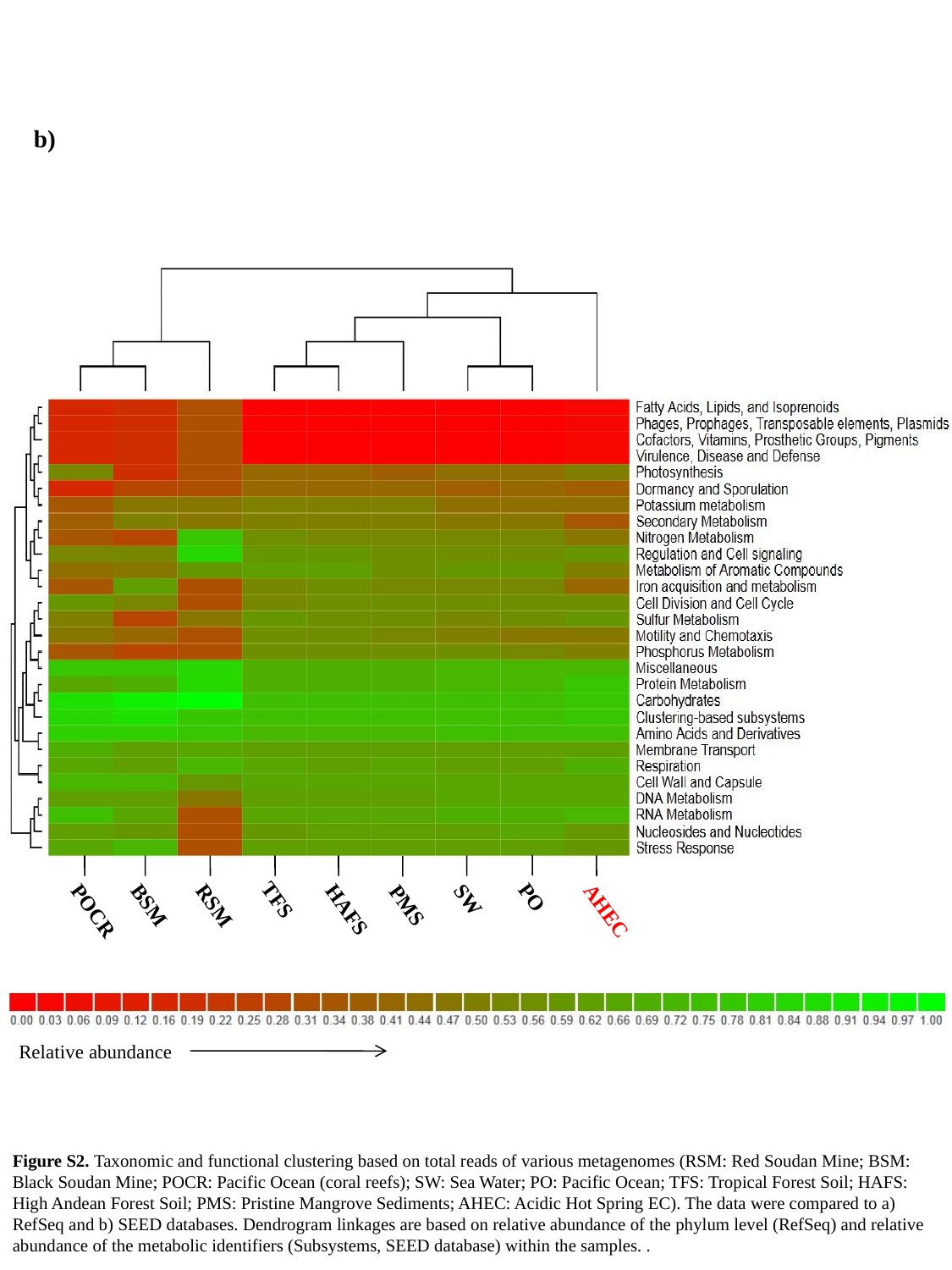

b)
PO
SW
TFS
BSM
PMS
RSM
HAFS
AHEC
POCR
Relative abundance
Figure S2. Taxonomic and functional clustering based on total reads of various metagenomes (RSM: Red Soudan Mine; BSM: Black Soudan Mine; POCR: Pacific Ocean (coral reefs); SW: Sea Water; PO: Pacific Ocean; TFS: Tropical Forest Soil; HAFS: High Andean Forest Soil; PMS: Pristine Mangrove Sediments; AHEC: Acidic Hot Spring EC). The data were compared to a) RefSeq and b) SEED databases. Dendrogram linkages are based on relative abundance of the phylum level (RefSeq) and relative abundance of the metabolic identifiers (Subsystems, SEED database) within the samples. .
