# Supplementary material for: Structural and Functional Insights from the Metagenome of an Acidic Hot Spring Microbial Planktonic Community in the Colombian Andes
Source: PLoS One. 2012 Dec 14;7(12):e52069. doi: 10.1371/journal.pone.0052069 (PMC3522619; doi:10.1371/journal.pone.0052069)
Supplement: Figure S3 — Partial a) nitrogen and b) sulfur pathways identified by KEGG affiliation of the sequences from EC hot spring. Boxes indicate the KEGG characteristic identified and numbers in gray circles indicate the amount of sequence reads affiliated to the KEGG function. (PPT) [file pone.0052069.s003.ppt]

## Slide 1
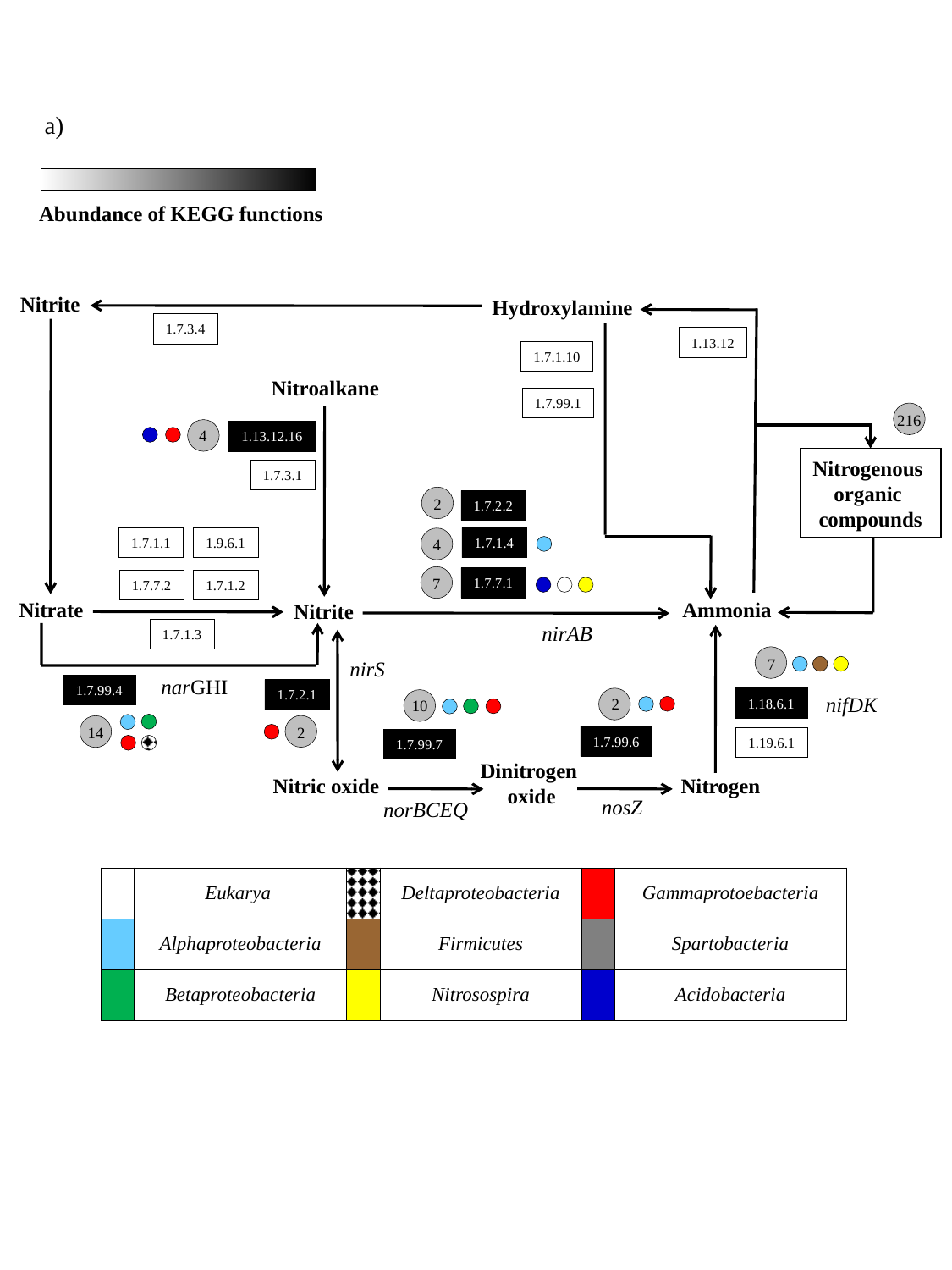

a)
Abundance of KEGG functions
Nitrite
Hydroxylamine
1.7.3.4
1.13.12
1.7.1.10
Nitroalkane
1.7.99.1
216
4
1.13.12.16
Nitrogenous
organic
compounds
1.7.3.1
2
1.7.2.2
1.7.1.1
1.9.6.1
1.7.1.4
4
7
1.7.7.1
1.7.7.2
1.7.1.2
Nitrate
Ammonia
Nitrite
nirAB
1.7.1.3
7
nirS
narGHI
1.7.99.4
1.7.2.1
nifDK
2
1.18.6.1
10
14
2
1.7.99.6
1.19.6.1
1.7.99.7
Dinitrogen
oxide
Nitric oxide
Nitrogen
nosZ
norBCEQ
| | Eukarya | | Deltaproteobacteria | | Gammaprotoebacteria |
| --- | --- | --- | --- | --- | --- |
| | Alphaproteobacteria | | Firmicutes | | Spartobacteria |
| | Betaproteobacteria | | Nitrosospira | | Acidobacteria |

## Slide 2
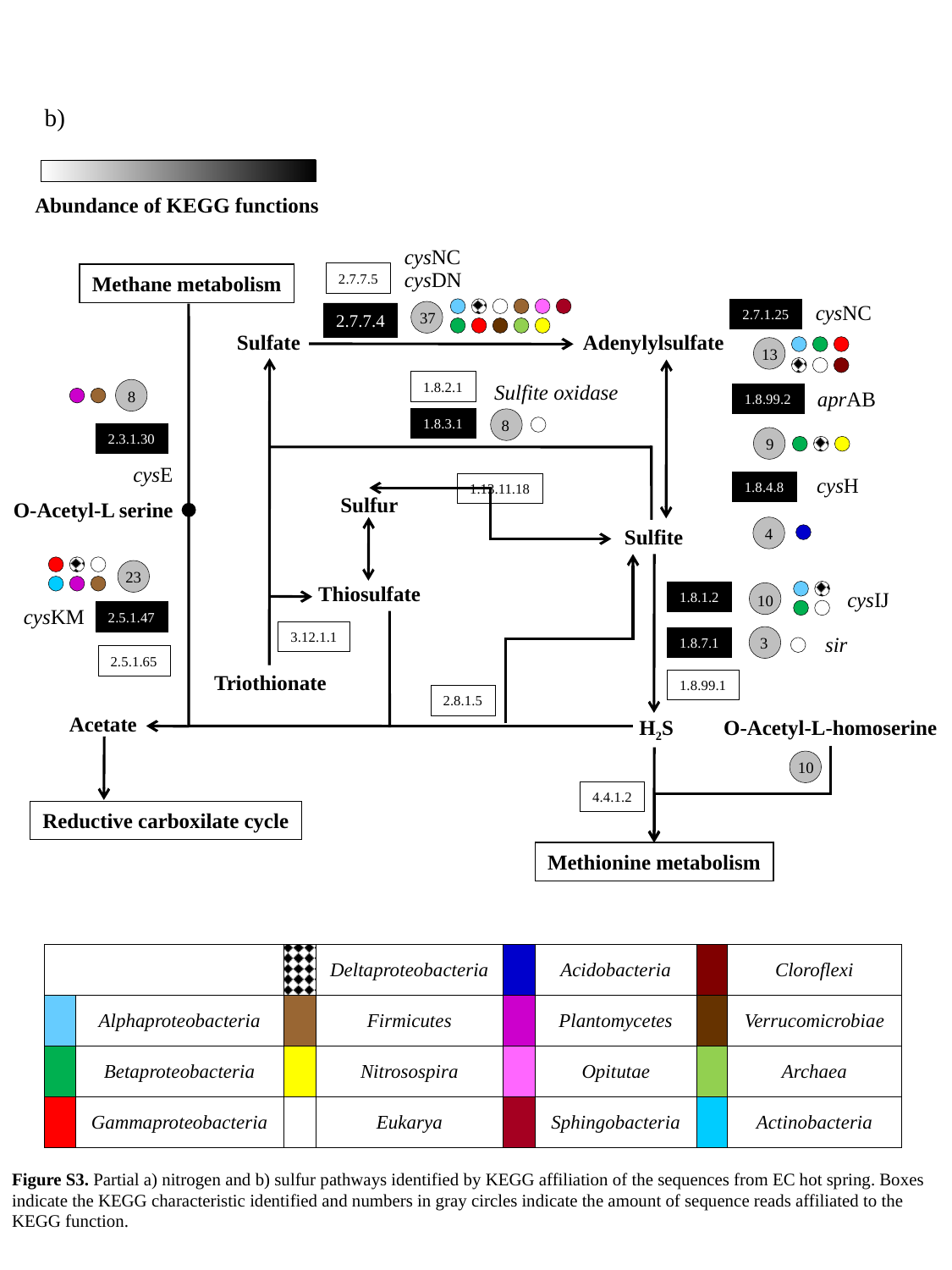

b)
Abundance of KEGG functions
cysNC
cysDN
2.7.7.5
Methane metabolism
cysNC
2.7.1.25
37
2.7.7.4
Sulfate
Adenylylsulfate
13
1.8.2.1
Sulfite oxidase
aprAB
8
1.8.99.2
1.8.3.1
8
2.3.1.30
9
cysE
cysH
1.8.4.8
1.13.11.18
Sulfur
O-Acetyl-L serine
Sulfite
4
23
10
Thiosulfate
cysIJ
21
1.8.1.2
10
cysKM
2.5.1.47
3.12.1.1
sir
3
1.8.7.1
2.5.1.65
Triothionate
1.8.99.1
2.8.1.5
Acetate
H2S
O-Acetyl-L-homoserine
10
4.4.1.2
Reductive carboxilate cycle
Methionine metabolism
| | | | Deltaproteobacteria | | Acidobacteria | | Cloroflexi |
| --- | --- | --- | --- | --- | --- | --- | --- |
| | Alphaproteobacteria | | Firmicutes | | Plantomycetes | | Verrucomicrobiae |
| | Betaproteobacteria | | Nitrosospira | | Opitutae | | Archaea |
| | Gammaproteobacteria | | Eukarya | | Sphingobacteria | | Actinobacteria |
Figure S3. Partial a) nitrogen and b) sulfur pathways identified by KEGG affiliation of the sequences from EC hot spring. Boxes indicate the KEGG characteristic identified and numbers in gray circles indicate the amount of sequence reads affiliated to the KEGG function.
